# Factors influencing the intention of the intensive care unit nurses to take preventive measures against deep vein thrombosis: a multicenter, cross sectional study

**DOI:** 10.3389/fmed.2025.1663913

**Published:** 2025-12-02

**Authors:** Jing Nie, Zhengying Jiang

**Affiliations:** Critical Care Medicine, Chongqing University Cancer Hospital, Chongqing, China

**Keywords:** nursing, venous thromboembolism, prevention, analysis of influencing factor, intention

## Abstract

**Purpose:**

To assess the current situation and factors influencing the ICU nurses’ intention to take measures to prevent deep vein thrombosis, and to provide a reference basis for developing strategies to intervene in the same.

**Methods:**

A questionnaire survey was conducted on 575 ICU nurses in 16 tertiary care hospitals across China, including a general information questionnaire and the Chinese Version of Nurses’ Intention to Use Deep Vein Thrombosis Preventive Measures Questionnaire (C-NUDQ), to assess the current situation of the ICU nurses’ intention to take preventive measures against deep vein thrombosis (DVT) and to analyse the influencing factors.

**Results:**

The total score of the ICU nurses’ intention to take measures to prevent DVT was (93.32 ± 12.62), of which the intention dimension score was (19.24 ± 2.84), behavioural attitude dimension score was (26.34 ± 3.37), subjective normative dimension score was (25.28 ± 3.89), and perceived behavioural control dimension score was (22.46 ± 4.70). The results of multiple linear regression analysis showed that education, years of ICU work, ICU specialist nurses, and they with training in knowledge related to DVT were the main influencing factors in the ICU nurses’ intention to take measures to prevent DVT.

**Conclusion:**

The intensive care unit nurses’ overall intention to adopt deep vein thrombosis preventive measures was at a moderately high level, and the remaining dimension scores that ranked from the highest to the lowest were for behavioural attitude, subjective norm, and perceived behavioural control. Education, years of intensive care unit work, they are intensive care unit specialist nurses, and they had received training in deep vein thrombosis-related knowledge were the influencing factors for the intensive care unit nurses’ intention to take preventive measures against deep vein thrombosis.

## Introduction

Deep vein thrombosis (DVT) is a disorder of venous return caused by abnormal blood coagulation in deep veins, often occurring in the lower extremities (1). At present, the primary treatment options are anticoagulation and thrombolysis therapy, as well as surgical embolectomy, etc. (1). Ahmad Kloub et al. (2) reported that the incidence of venous thromboembolism in Intensive Care Unit (ICU) patients was as high as 3%. The annual number of new cases exceeds 1 million in the United States and is about 1.5 million in Europe (3). According to the results of a cross-sectional survey study (4), the incidence of DVT in patients admitted to the ICU in China was 13.6%, among them, 71% were intermuscular venous thrombosis.

The importance of minimizing patient exposure to high-risk thrombotic factors to improve the prevention of DVT has been noted in several guidelines (5). Therefore, prevention of DVT is an effective method for reducing the morbidity and mortality in critically ill patients in the ICU. However，preventive care for DVT has not received adequate attention, in clinical practice, there are still a number of patients at high risk of lower extremity deep vein thrombosis who have not received targeted preventive interventions (6).

The theory of planned behaviour (TPB) is a social cognitive theory widely used in behaviour interventions and predictions. TPB theory suggests that an individual’s behavioural intention is the most direct and critical factor that affects the behaviour of the individual and self-intention; meanwhile, it is directly influenced by factors such as behavioural attitudes, subjective norms, and perceived behavioural control of the individual who performs the behaviour (7). Therefore, the prevention of DVT requires a detailed understanding of nurses’ intention to take preventive measures against DVT in the ICU and its influencing factors. We aimed to investigate this intention with a view to providing a reference for further the development of new targeted intervention strategies.

## The study

### Aim and objective

This study aimed to introduction of a new assessment tool for China to evaluate the intention of ICU nurses to take measures to prevent deep vein thrombosis. To understand the factors influencing the intention of ICU nurses to take preventive measures against deep vein thrombosis (DVT), and to provide a reference for developing intervention strategies to strengthen the intention of ICU nurses to take preventive measures against DVT.

### Design and methods

#### Research subjects

The Chinese Version of Nurses’ Intention to Use Deep Vein Thrombosis Preventive Measures Questionnaire(C-NUDQ) (8) includes 15items, 4open-ended questions, and the general information includes 10 questions, so there are 19 variables in the questionnaire. The final sample size is calculated according to the calculation method proposed by Kendall (9), that is, one item corresponds to 5–10 sample sizes. Considering the existence of invalid questionnaires in sample recovery, expanding the sample size by 10–20% would require an estimated 319–348 questionnaires. We divided China into four regions: east, south, west, and north, from October 2021 to December 2021, ICU nurses meeting the inclusion and exclusion criteria in 16 provincial and municipal hospitals in China were selected by convenient sampling method. The specific investigation work of each hospital was completed by their head nurses. Through preliminary research, we found that nurses from these 16 hospitals could meet the sample size requirements of our article. Inclusion criteria were as follows: ① Nurses with nursing certification and working in ICU. ② Age ≥18 years old. Exclusion criteria were as follow: ① less than 1 year of ICU participation. ② Nurses from other hospitals who come to our hospital for further study, nurses undergoing standardized training, or internships. All nurses signed an informed consent form before participating in the study.

#### Research methods and tools

##### General information questionnaire

The general information questionnaire, self-designed based on literature review, included items such as gender, age, work experience, professional title, position, educational qualification, and whether they had received training on DVT.

##### Chinese version of the NUDQ

**Figure 1:**
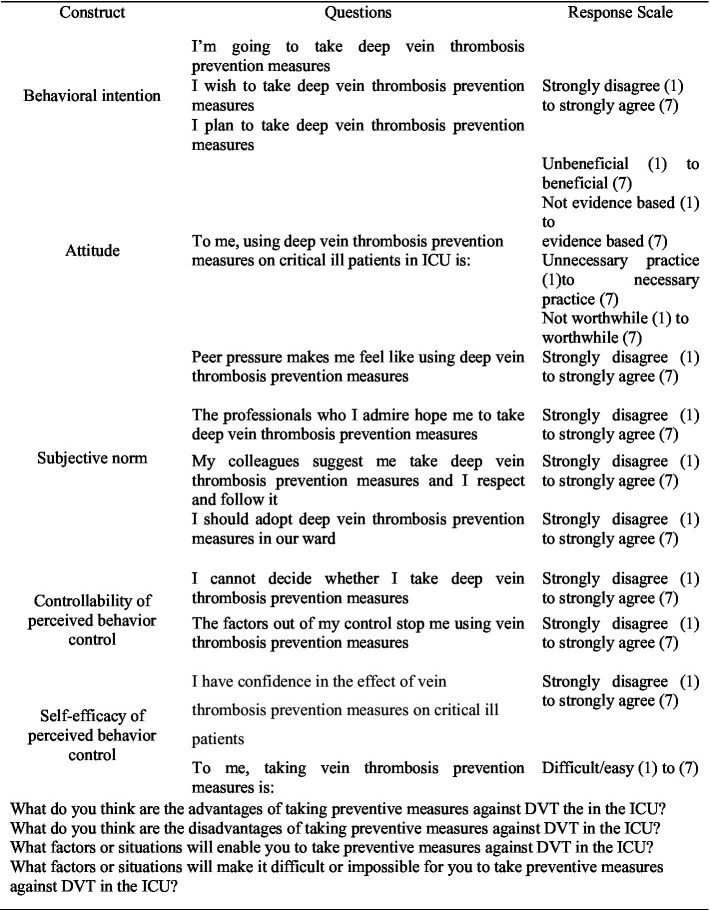
The Chinese version of nurses’ intention to use deep vein thrombosis preventive measures questionnaire.

The scale was developed in 2020 by Dr. Mona Ibrahim Hebeshy and her team at Kent State University based on TPB theory, and has been verified with good reliability and validity (10). After obtaining the original author’s authorization, our research group strictly followed the Brislin translation model, to translate and back-translate the scale. We invited 16 experts to evaluate the validity of the scale’s content, and obtained the Chinese version of the scale. At the same time, this scale was used to assess the intention of 674 ICU nurses to take preventive measures against deep vein thrombosis, and the reliability and validity of the scale were tested (8) (see Figure 1). Results of the reliability and validity testing showed that the total Cronbach’s α coefficient of the scale was 0.872, the split-half coefficient was 0.840, Cronbach’s α coefficient of each dimension ranged between 0.735–0.926, and the test-retest reliability was 0.843. Results of confirmatory factor analysis showed that the data had fair goodness of fit, indicating that C-NUDQ was suitable as a tool to evaluate the intention of ICU nurses in China to take preventive measures against DVT. The C-NUDQ scale consists of four dimensions, 15items, and four open-ended questions. The four dimensions are behavioural intention, behavioural attitudes, subjective norms, and perceived behavioural control. All 15 items were scored on a 7-point Likert scale, and each item was scored from 1 to 7 points. The scores of all items related to each dimension were pooled, and the average score of each dimension was calculated. The higher the score on the behavioural intention items, the stronger the intention to take preventive measures against DVT. The total score of the behavioural intention dimension ranged from 3 to 21 points, of which 3 to 8 points represented a low level, 9 to 14 points represented a medium level, and 15 to21 points represented a high level. The higher the score, the stronger the intention of ICU nurses to take preventive measures against DVT. Four open-ended questions assessed the advantages and disadvantages of taking preventive measures against DVT, as well as the factors and barriers that affect nurses’ intention to take DVT preventive measures in the ICU. These questions were as follow:

Open-ended question 1: What do you think are the advantages of taking preventive measures against DVT the in the ICU?

Open-ended question 2: What do you think are the disadvantages of taking preventive measures against DVT in the ICU?

Open-ended question 3: What factors or situations will enable you to take preventive measures against DVT in the ICU?

Open-ended question 4: What factors or situations will make it difficult or impossible for you to take preventive measures against DVT in the ICU?

### Data collection

In this study, the electronic questionnaires on wjx.cn were first imported into WeChat, and then distributed and collected through the designated sharing of wjx.cn questionnaires on WeChat. Prior to the distribution of the electronic questionnaires, the research team obtained approval from the ethics committee of the investigated hospitals, and this study was approved and supported by the corresponding hospital leadership teams.

### Ethical approval

The study involving humans were approved by the Ethics Committee of the Hospital. Before conducting the survey, we fully explained the study objectives and survey instructions to the ICU nurses. ICU nurses could voluntarily participate in this study. If they agreed to participate, they signed an informed consent form, and they could withdraw from the study at any time. All questionnaires were completed anonymously, and the data collected were used solely for the purpose of this study. The basic information of the participating ICU nurses was kept confidential.

### Statistical methods

All the questionnaire data were analysed using SPSS 26.0 (IBM Corp., Armonk, NY, USA). For descriptive statistics, continuous variables (e.g., age) and categorical variables (e.g., gender) were expressed as frequencies and percentages. An independent samples t-test and one-way ANOVA were used to compare the differences based on general information (sex, education level, and previous training on knowledge of DVT) in terms of intention, and *p* < 0.05 was considered to indicate statistically significant difference. The total score of the C-NUDQ scale was used as the dependent variable, and the statistically significant variables in univariate analysis were used as the independent variables for multiple linear regression analysis.

## Results

### General demographics

In this study, we distributed 600 questionnaires and returned 575 questionnaires, with a return rate of 95.80%. Among all the participating nurses, 105 were male (18.26%), 470 were female (81.74%), 449 (78.09%) had a Bachelor’s degree or above, 34.26% of the nurses had worked in the ICU for 6–10 years, 50.44% of them were senior nurses, most of them were contract nurses (83.09%), and 79.3% of the nursing staff had received training on DVT (see Table 1).

**Table 1:** Basic information of research subjects (*n* = 575).

Variable	Class	*N*	Proportion (%)
Gender	Male	105	18.26
	Female	470	81.74
Age group (years)	20~	271	47.13
	30~	278	47.35
	>40	26	4.52
Educational qualification	College and lower	126	21.91
	Bachelor’s degree	424	73.74
	Master’s degree and higher	25	4.35
Professional Title	Nurse	121	21.04
	Senior nurse	290	50.44
	Supervisor nurse	152	26.43
	Deputy chief nurse and above	12	2.09
Designation	Nurse	493	85.74
	Nursing team leader	64	11.13
	Deputy head nurse and above	18	3.13
Duration of overall work experience(years)	<3	105	18.26
	3~	105	18.26
	6~	99	17.22
	9~	98	16.04
	≥11	168	29.22
Duration of ICU work experience(years)	<3	68	11.83
	3~	78	13.57
	6~	157	27.30
	9~	149	25.91
	≥11	123	21.39
Type of employment	Authorized	96	16.70
	Contractual	479	83.30
Whether a specialized ICU nurse	No	279	48.52
	Yes	296	51.48
Hospital type	General hospital	326	56.70
	Specialised hospital	249	43.30
ICU type	Comprehensive ICU	522	90.78
	ICU Emergency	23	4.00
	Other	30	5.22

ICU, intensive care unit.

### Scores on different dimensions of the C-NUDQ

This study strictly adhered to the framework of the Theory of Planned Behaviour (TPB), and measured the intention of ICU nurses to take DVT prevention measures, rather than directly observing their actual behaviours. The ICU nurses scored (19.24 ± 2.84) on the behavioural intention dimension to take preventive measures against DVT, (26.34 ± 3.37) on the behaviour attitude dimension, (25.28 ± 3.89) on the subjective norm dimension, and (22.46 ± 4.70) on the perceived behavioural control dimension. See Table 2 for details.

**Table 2:** Scores of nurses’ intention to take preventive measures against DVT (*n* = 575).

Variable	Number of items	Score range	Score	Average score of items
Total score on the intention scale	15	25 ~ 105	93.32 ± 12.62	6.22 ± 0.84
Behavioural intention	3	3 ~ 21	19.24 ± 2.84	6.41 ± 0.95
Behavioural attitudes	4	4 ~ 28	26.34 ± 3.37	6.59 ± 0.84
Subjective norms	4	6 ~ 28	25.28 ± 3.89	6.32 ± 0.97
Perceived behavioural control	4	11 ~ 28	22.46 ± 4.70	5.61 ± 1.17

### Univariate analysis of factors influencing nurses’ intention to take preventive measures against DVT

One-way ANOVA was performed with general information of ICU nurses, such as sex and age, as the independent variables, and the C-NUDQ total score as the dependent variable. Results showed that educational qualification, work experience in the ICU, the nurse was an ICU nurse specialist, the presence of prevention standards for DVT, and training had been received on DVT had influences on the intention to take preventive measures, with statistically significant differences (*p* < 0.05). See Table 3 for details.

**Table 3:** Comparison of scores of nurses’ intention to take preventive measures against DVT among nurses according to demographic characteristics (*n* = 575).

Item	Category	Number of subjects (%)	Score	*F/t*	*p*
Gender	Male	105 (18.26)	95.03 ± 12.38	2.359	0.125
	Female	470 (81.74)	92.94 ± 12.66		
Age group (years)	20~	271 (47.13)	94.24 ± 11.60	1.392	0.244
	30~	278 (47.35)	92.23 ± 13.76		
	>40	26 (4.52)	95.35 ± 9.57		
Educational qualification	College and lower	126(21.91)	85.95 ± 16.78	60.407	0.000
	Bachelor’s degree	424 (73.74)	95.34 ± 8.79		
	Master’s degree and higher	25 (4.35)	105.00 ± 0.00		
Professional Title	Nurse	121 (21.04)	93.50 ± 13.42	0.978	0.419
	Senior nurse	290 (50.44)	93.61 ± 12.38		
	Supervisor nurse	152 (26.43)	92.18 ± 12.79		
	Deputy head nurse and above	12 (2.09)	98.73 ± 4.92		
Designation	Nurse	493 (85.74)	92.89 ± 12.86	1.767	0.152
	Nursing team leader	64 (11.13)	95.06 ± 11.84		
	Deputy head nurse and above	18 (3.13)	100.14 ± 5.08		
Duration of overall work experience(years)	<3	105 (18.26)	94.61 ± 11.76	0.627	0.643
	3~	105 (18.26)	93.95 ± 9.71		
	6~	99 (17.22)	92.37 ± 13.95		
	9~	98 (16.04)	92.34 ± 13.76		
	≥11	168 (29.22)	93.25 ± 13.28		
Duration of ICU work experience(years)	<3	68 (11.83)	81.74 ± 22.37	22.538	0.000
	3~	78 (13.57)	94.14 ± 8.34		
	6~	157 (27.30)	92.10 ± 9.53		
	9~	149 (25.91)	96.66 ± 10.01		
	≥11 years	123 (21.39)	96.71 ± 9.52		
Type of employment	Authorized	96 (16.70)	92.28 ± 13.20	0.396	0.674
	Contractual	479 (83.30)	93.53 ± 12.51		
Whether a specialized ICU nurse	Yes	296 (51.48)	96.36 ± 10.02	37.770	0.000
	No	279 (48.52)	90.09 ± 14.22		
Hospital type	General hospital	326 (56.70)	93.42 ± 12.68	0.045	0.833
	Specialised hospital	249 (43.30)	93.19 ± 12.58		
ICU type	Comprehensive ICU	522 (90.78)	93.38 ± 12.57	0.322	0.810
	ICU Emergency	23 (4.40)	93.30 ± 13.61		
	Other	30(5.22)	91.90 ± 13.16		
have prevention standards for DVT	Yes	553 (96.17)	93.70 ± 12.08	13.109	0.000
	No	22 (3.83)	83.86 ± 20.53		
have received training on knowledge of DVT	Yes	433 (79.30)	98.53 ± 5.92	621.147	0.000
	No	142 (20.70)	77.44 ± 14.27		

ICU, intensive care unit; DVT, deep vein thrombosis.

### Multiple linear stepwise regression analysis of factors influencing nurses’ intention to take preventive measures against DVT

Based to the results of independent samples t-test and one-way ANOVA, multivariate stepwise regression analysis was performed with the C-NUDQ score as the dependent variable, and five factors (educational qualification, work experience in the ICU, whether the nurse is an ICU nurse specialist, presence/absence of prevention standards for DVT, and whether the nurse had received training on DVT) as the independent variables. The significance levels for inclusion and exclusion of independent variables were α_in_ = 0.05, α_out_ = 0.10, respectively, and the specific values assigned to the independent variables are shown in Table 4. In the present study, the results indicated that educational qualification, work experience in the ICU, the nurse was an ICU nurse specialist, and with training on DVT had been received were the main factors influencing nurses’ intention to take DVT preventive measures. There were four variables included in the regression equation, which accounted for 54% of the variation in nurses’ intention to take preventive measures against DVT (*F* = 257, *p* = 00, R = 43, R^2^ = 40). The details are shown in Tables 4, 5.

**Table 4:** Value assignment of independent variables in the multiple linear regression analysis of nurses’ intention to take DVT preventive measures (*n* = 575).

Independent variable	Value assignment
Educational qualification	Technical secondary school = 1, junior college = 2, Bachelor’s degree = 3, Master’s degree or above = 4.
Work experience in the ICU (years)	<3 = 1, 3–5 = 2, 6–8 = 3, 9–10 = 4, ≥ 11 = 5
ICU specialist nurse	No = 0, yes = 1
Presence of prevention standards for DVT	No = 0, yes = 1
raining on DVT had been received	No = 0, yes = 1

ICU, intensive care unit; DVT, deep vein thrombosis.

**Table 5:** Multiple linear regression of nurses’ intention to take DVT preventive measures.

Independent variable	Non-standardised coefficient	Standard error (SE)	Standardised coefficient	*t-*value	*p-*value
	(B value)		ꞵ value		
(Constant)	97.293	2.858	—	34.046	0.000
having received relevant training	−18.151	0.807	-0.621	−22.495	0.000
Educational qualification	5.910	0.689	0.241	8.577	0.000
Work experience in the ICU	1.241	0.275	0.125	4.513	0.000
ICU specialist nurse	−1.396	0.699	−0.055	−1.998	0.046

ICU, intensive care unit; DVT, deep vein thrombosis.

### Answers to open-ended questions

In terms of answers to the open-ended questions, nurses reported that the most common advantages of taking DVT preventive measures in critically ill patients included reducing the risk of pulmonary embolism, reducing the length of stay in the ICU, reducing the morbidity and mortality of cardiovascular diseases, and reducing medical costs. On the other hand, the nurses identified the possibility of bleeding, time-consuming nature of preventative measures, increased staff workload, and possible harm to patients as the most common disadvantages. In addition, some nurses answered that the use of an air pressure pump or elastic compression socks could help facilitate preventive measures against DVT in the ICU. Many nurses mentioned the importance of nurses’ knowledge and skills in preventing DVT, which is an important factor in enabling nurses to take preventive measures against DVT in the ICU. Moreover, 95 nurses (16.5%) expressed that training programs, workshops, and seminars would help prevent DVT; Eighty-seven nurses (15.1%) stated that they believed that patients being too ill, for example, patients with subarachnoid haemorrhage, lower limb surgeries, or fractures, was the main barrier to providing drug prophylaxis in the ICU. On the other hand, the patients being in a coma or with delirium would also limit the adoption of preventive measures. Ten head nurses (1.7%) expressed that their workload and staff shortages, coupled with emergency situations and the pressure of working in the ICU, also led to difficulties.

## Discussion

Studies have shown that damage to the venous vessel wall, altered blood composition, and abnormal blood rheology are the three major influencing factors for thrombosis (1). For patients with potential high-risk factors for thrombosis, such as advanced age, long-term bed rest, post-surgical braking, pregnancy, and hypercoagulable blood, oral anticoagulants and dissipative drugs and regular active or passive (manual or mechanical) limb exercises, limb elevation, and early rehabilitation exercises are recommended to enhance blood return and thus reduce the risk of thrombosis. According to the survey results, the current status of DVT preventive care for inpatients is not optimistic (6), and the existing studies mainly focus on DVT monitoring, patient education, and risk assessment, the groups of nurses investigated and studied are mostly in the operating room, orthopedics and obstetrics and gynecology, but less in the ICU nurses (11–13), and most of them focus on the investigation of nurses’ knowledge, belief and behaviour (14, 15), and lack of research on intention. Wang Y’s study found that the implementation of an integrated care model combined with health education in patients with hip arthroplasty is beneficial to improving self-efficacy, patient trauma coping style, promoting early hip function recovery and improving nursing care satisfaction (16). Senay Yohannes et al. used a structured pretested self-administered questionnaire to assess the knowledge, practice, and associated factors towards deep venous thrombosis prevention among nurses working at Amhara region comprehensive specialized hospitals, Northwest, Ethiopia. Knowledge and practice of the nurses regarding the prevention of deep venous thrombosis were found to be inadequate (17). This study investigated the willingness of ICU nurses to take preventive measures against deep vein thrombosis and the influencing factors.

### Current status of nurses’ intention and influencing factor to take preventive measures against DVT

The survey results of 575 ICU nurses demonstrated a score of 19.24 ± 2.84 points on the dimension of behavioural intention to take preventive measures against DVT, suggesting a relatively strong intention to take preventive measures. Consistent with the results of the original authors (10). The score was 26.34 ± 3.37 for the behaviour attitude dimension, 25.28 ± 3.89 for the subjective norm dimension, and 22.46 ± 4.70 for the perceived behavioural control dimension. The results of this study revealed that educational qualification, work experience in the ICU, ICU nurse specialist, and the nurse had received training on DVT were the factors influencing nurses’ intention to take DVT preventive measures.

### Higher educational qualification and richer ICU work experience was associated with stronger intention to take preventive measures

This study shows that the Bachelor’s degree score is 95.34 ± 8.79, Master’s degree and higher score is 105.00 ± 0.00. The results revealed that the higher the educational qualification of nurses, the stronger their intention to take preventive measures against DVT, which might be related to the fact that nurses with a relatively high educational qualification and richer work experience in the ICU had a broader knowledge base and more working experience. This is consistent with the research results of Li Jiaxin et al. (18). These nurses were relatively adept at professional skills, could better deal with complex clinical problems, and had a greater sense of confidence and self-efficacy. Generally, ICU nurses are more willing to engage in behaviours that they are interested in and capable of performing. Yao et al.’s survey results showed that the influence of educational level on the knowledge and prevention practice level of nurses regarding the risk of deep vein thrombosis (19). The reason might be that nurses with higher educational qualification have higher demands for self-development or professional growth and are more eager to improve their clinical nursing skills. Therefore, in future training or nursing management, we should pay attention to young nurses with relatively lower educational qualification and limited work experience in the ICU.

### ICU nurse specialists had higher behavioural intention

The results indicated that one of the positive influencing factors was that they were an ICU nurse specialist. ICU nurse specialists have more opportunities to receive relevant training, and they may have a stronger drive for autonomous learning and higher expectations. According to the theory of reasoned action, an individual’s positive experience in the past will strengthen their behavioural intention. Thus, the experiences of ICU nurse specialists in preventing DVT, and the skills and confidence established in preventing DVT, may promote their positive behavioural intention.

### ICU nurses who had received training on DVT had higher behavioural intention

The results of this study indicated that having received insufficient training on DVT was a major barrier to the implementation of preventive measures. This is possibly because nurses who have received training have more knowledge and confidence in taking DVT preventive measures, and training has changed nurses’ knowledge, beliefs, and attitudes towards the prevention of DVT. The training can be delivered via a variety of methods, for example, a combination of online and offline instruction and team teaching. Guidelines for prevention of DVT should form the basis for the training. After the training, the training quality should be checked regularly to increase nurses’ awareness of taking preventive measures against thrombosis.

### About open-ended questions

The findings of the open-ended questions showed that the ICU nurses endorsed a number of beliefs. For example, the attitudinal beliefs pertaining to the advantages of using DVT preventive measures reflected concerns about the reduced risks of pulmonary embolism, decreased length of ICU stay, decreased rates of morbidity and mortality, and improved cardiovascular benefits. Nurses believed that barriers preventing the use of DVT preventive measures in the ICU are the risks for some patients and instability of patients’ conditions. In addition, the ICU population includes patients with complex medical comorbidities, post-operative general surgical patients, poly-trauma, and neuro-intensive care as well as bariatric patients coupled with risk factors acquired during their ICU stays.

### Limitations

There are several limitations in our study. The main limitation is the use of convenience sampling in this study will affect the representativeness and generalizability of the findings. Second, the study focuses solely on intention and potential self-report, no data on actual DVT prevention practices, which may introduce social desirability bias or recall bias. To minimize the impact of bias to the greatest extent, we selected nurses from various regions in China (east, west, south, and north) to participate, and the participating group included nurses of different age groups, different educational backgrounds, and different ICU working experiences. Third, in the current study, the findings showed that the scale had good reliability and validity, but it was not validated in other countries and may need further testing and refinement. Fourth, the hypothesis that DVT-trained nurses would have more knowledge, confidence, etc. was not tested in this study. Fifth, we conducted the sample selection by surveying only the nursing staff in tertiary care hospitals and did not take other types of hospitals into consideration.

### Recommendations or implications for practice and/or further research

Future research should include larger sample sizes and test the applicability of the C-NUDQ scale among ICU nurses in different regions. At the same time, quantitative research can be conducted to understand the current implementation status and influencing factors of nurses’ measures to prevent deep vein thrombosis. Nursing managers should increase matching nursing staff, so that preventive measures can be effectively implemented, and establish a series of rules and regulations and evaluation mechanism in clinical, to ensure the rational operation of the system and norms, improve the intention of clinical nurses to take measures to prevent deep vein thrombosis, so that the prevention and treatment of deep vein thrombosis can be effective.

## Conclusion

The investigation of this study revealed that education, years of ICU work, they were ICU specialist nurses, and they had received training on knowledge related to deep vein thrombosis were the main influencing factors on the ICU nurses’ intention to take measures to prevent deep vein thrombosis. When taking measures to prevent lower extremity DVT formation in the future, nursing managers should increase training related to DVT prevention and treatment for nurses, especially those with lower education and fewer years of ICU work, to improve the nurses’ knowledge of DVT and complications. To effectively improve the preventive measures of lower limb DVT, the incidence of lower limb DVT should be reduced and the quality of life of patients improved.

## Data Availability

The original contributions presented in the study are included in the article/supplementary material, further inquiries can be directed to the corresponding author.
